# The Inclusive Professional Framework for Societies: Changing Mental Models to Promote Diverse, Equitable, and Inclusive STEM Systems Change

**DOI:** 10.3389/fsoc.2021.784399

**Published:** 2022-02-18

**Authors:** Gretalyn M. Leibnitz, Donald L. Gillian-Daniel, Robin M c C. Greenler, Rebecca Campbell-Montalvo, Heather Metcalf, Verónica A. Segarra, Jan W. Peters, Shannon Patton, Andrea Lucy-Putwen, Ershela L. Sims

**Affiliations:** ^1^ Amplifying the Alliance to Catalyze Change for Equity in STEM Success (ACCESS+), Washington, DC, United States; ^2^ ProActualize Consulting, LLC, Moscow, ID, United States; ^3^ University of Wisconsin-Madison, Madison, WI, United States; ^4^ INCLUDES Aspire Alliance, Madison, WI, United States; ^5^ University of Connecticut, Mansfield, CT, United States; ^6^ Women in Engineering, ProActive Network, Washington, DC, United States; ^7^ High Point University, High Point, NC, United States; ^8^ Katalytik, Christchurch, United Kingdom

**Keywords:** inclusive professional framework for societies, mental models, intercultural mindfulness, equity mindset, inclusive relationships, identity awareness, influential actions, DEI (or Diversity Equity and Inclusion)

## Abstract

Science, technology, engineering, and mathematics (STEM) professional societies (ProSs) are uniquely positioned to foster national-level diversity, equity, and inclusion (DEI) reform. ProSs serve broad memberships, define disciplinary norms and culture, and inform accrediting bodies and thus provide critical levers for systems change. STEM ProSs could be instrumental in achieving the DEI system reform necessary to optimize engagement of all STEM talent, leveraging disciplinary excellence resulting from diverse teams. Inclusive STEM system reform requires that underlying “mental models” be examined. The Inclusive Professional Framework for Societies (*IPF: Societies*) is an interrelated set of strategies that can help ProSs change leaders (i.e., “boundary spanners”) and organizations identify and address mental models hindering DEI reform. The *IPF: Societies* uses four “I's”—Identity awareness and Intercultural mindfulness (i.e., equity mindset) upon which inclusive relationships and Influential DEI actions are scaffolded. We discuss how the *IPF: Societies* complements existing DEI tools (e.g., *Women in Engineering ProActive Network's* Framework for Promoting Gender Equity within Organization; *Amplifying the Alliance to Catalyze Change for Equity in STEM Success'* Equity Environmental Scan Tool). We explain how the *IPF: Societies* can be applied to existing ProS policy and practice associated with common ProS functions (e.g., leadership, membership, conferences, awards, and professional development). The next steps are to pilot the *IPF: Societies* with a cohort of STEM ProSs. Ultimately, the *IPF: Societies* has potential to promote more efficient, effective, and lasting DEI organizational transformation and contribute to inclusive STEM disciplinary excellence.

## Inclusive Stem Disciplinary Excellence Requires Systems Reform

Addressing complex global challenges, such as climate change and health disparities, requires optimal engagement of people trained in science, technology, engineering, and mathematics (STEM). Because diverse teams embody enhanced capacity for problem solving, innovation, and resilience, they advance disciplinary excellence in a way that homogenous groups cannot (e.g., Page, 2007; Borman et al., 2010; Page, 2017; McGee, 2020). Consequently, not only are more STEM-trained people needed, but specifically more diverse STEM-trained people are needed.

Unfortunately, STEM cultures often discourage diversity by reproducing exclusionary norms and values (Tonso, 1996, 1999, 2007; Seymour and Hewitt, 1997; Pawley and Tonso, 2011; BaillieKabo and Reader, 2012; Riley et al., 2014; Cech and Rothwell, 2018; Hughes, 2018; McGee, 2020). Current US STEM systems privilege white, and/or men in STEM, and are typically perceived as unwelcoming by marginalized groups, especially women from various backgrounds (Metcalf et al., 2018; McGee, 2020; Campbell-Montalvo et al., 2021a). Expression of majority priority is manifest in complex ways, such as equating masculinity with technical ability, embracing and centering whiteness (Hacker, 1981, 1989; Eisenhart and Finkel, 1998; Lohan and Faulkner, 2004; Faulkner, 2007; Foor et al., 2007; Tonso, 2007; Pawley and Tonso, 2011; BaillieKabo and Reader, 2012), and fostering the false idea that STEM is an apolitical, value-free, empirical meritocracy (McGee, 2020; Metcalf, 2017). Systems of power, privilege, and oppression intersect with those shaped by gender, race, ethnicity, sexuality, disability, nationality, class, and more (Crenshaw, 1989; Crenshaw 1991; Griffin and Museus, 2011; Collins, 2015; Metcalf, 2016; Warner et al., 2016; Metcalf et al., 2018). Collectively, these intersecting systems influence opportunities, create barriers, and can in turn promote exclusionary experiences for a variety of individuals, including women and other groups underrepresented in STEM. These experiences of exclusionary STEM systems are often replicated in, and sustained, not just in established STEM work environments but also by a STEM education system that socializes the next generation of the STEM workforce to abide by and reproduce these norms and values (Trouillot, 1995; Foucault, 2007; Tonso, 2007; Tonso, 2014).

While STEM systems reform is clearly needed to attract, retain, and support a thriving diverse STEM talent pool, there is widespread expectation that minoritized and marginalized people will, and should be, the ones tasked with changing a system by which they are oppressed and largely excluded (Forrester, 2020). Majoritized people receive disproportionate power within the current system, so it is incumbent on them to be leaders in STEM system change to promote inclusive disciplinary excellence. This change must be supported through both “intentional introspection and subsequent action” (Chaudhary and Berhe, 2020, pg. 3).

## Uncovering Professional Society Mental Models

Mental models are “deeply held beliefs and assumptions, and taken-for-granted ways of operating that influence how we think, what we do, and how we talk” (Kania et al., 2018, p. 4). We argue that intentional introspection of mental models can foster systems change. Systems change is “shifting the conditions that are holding the problem in place” (Kania et al., 2018). Kania et al. (2018) identified six conditions of systems change that are explicit (i.e., *policies*, *practices*, *resource flows*), semi-explicit (i.e., *relationships and connections*, *power dynamics*), and implicit (i.e., mental models). *Mental models* hold the other conditions in place. Unless we learn to work at the mental models level, other structural changes “...will, at best, be temporary or incomplete” (Kania et al., 2018, p.8). While work addressing mental models has been increasing in academic institutions (e.g., NSF ADVANCE-funded initiatives) and industry settings, few projects have undertaken these efforts within professional societies (ProSs).

Given the multiple, varied disciplinary functions performed by STEM ProSs, and that STEM ProSs often engage other STEM system gatekeepers (e.g., corporate, laboratory, and academic organizations), STEM ProSs are uniquely positioned as critical levers for STEM systems change (e.g., National Academy of Sciences et al., 2005). Peters and others (in press) identify 11 functions performed by STEM ProSs (i.e., governance and leadership; membership; programming; professionalization; student chapters; prizes, awards, and funding; outreach and engagement; employment; advocacy; and publishing). Through functions such as these, the ProS reinforces mental models regarding how the discipline “looks, feels, and acts.” Leaders are identified, innovations celebrated, and the next generation is nurtured. For example, students enter STEM degree programs with varying levels of social capital (Skvoretz et al., 2020), and ProSs keep them in their programs (Smith et al., 2021; Campbell-Montalvo et al., in press). Some STEM ProSs are actively engaged in STEM systems change to promote diversity, equity, and inclusion (DEI) through STEM ProS functions (e.g., Segarra et al., 2020a; Segarra et al., 2020b; Campbell-Montalvo et al., in press, Campbell-Montalvo et al., 2020; Etson et al., 2021). However, we believe that to foster greater engagement by STEM ProSs, more STEM ProS-specific tools are needed, especially those that can help make explicit and reframe mental models underpinning STEM ProS functions.

## The IPF: Societies as a Tool for Mental Model Changes

We offer the *Inclusive Professional Framework for Disciplinary and Professional Societies* (*IPF*: *Societies*) as an approach to help elucidate and adjust mental models that underlie STEM ProS functions (INCLUDES Aspire Alliance National Change, n.d.). The *IPF: Societies* is a framework that can be used to explore how internal conditions support and hinder current ProS DEI aspirations and help set a foundation for lasting organizational change. Specifically, the *IPF: Societies* is a research-informed approach that focuses on awareness and skill development to build an equity mindset—an orientation in which actions are grounded in understanding of how social positionings affect access to resources. This mindset creates greater capacity for inclusive relationships and supporting actions that are focused on DEI change. The *IPF: Societies* includes the four “I's”:

 1. Identity awareness,
 2. Intercultural mindfulness,
 3. Inclusive relationships, and
 4. Influential DEI actions.

**FIGURE 1:**
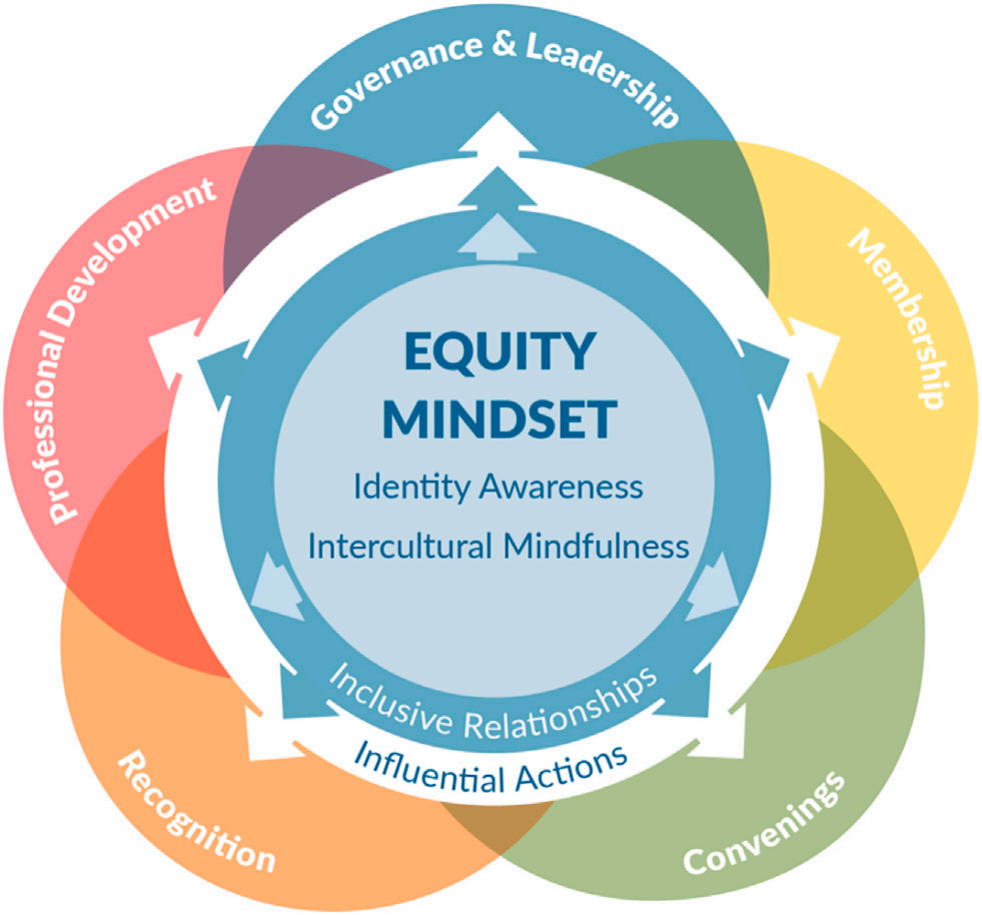
The IPF: Societies graphic with five example professional society functions (INCLUDES Aspire Alliance National Change, n.d.).

The *IPF: Societies* derives from the *Inclusive Professional Framework for Faculty* (*IPF: Faculty*). The *IPF: Faculty* was developed by the Aspire Alliance's National Change Initiative, which is part of the National Science Foundation's Inclusion across the Nation of Communities of Learners of Underrepresented Discoverers in Engineering and Science (NSF INCLUDES). The IPF: Societies was developed with input from leaders from the NSF ADVANCE-funded Amplifying the Alliance to Catalyze Change for Equity in STEM Success (ACCESS+), (n.d) Initiative, whose mission is to “accelerate the awareness, adoption, and adaptation of NSF ADVANCE evidence based, gender-related, DEI policies, practices, and programs within and across STEM ProSs, by providing support to STEM ProS boundary spanners.” Through partnership with ACCESS+ the *IPF: Societies* graphic was created, along with example functions (see Figure 1); and the model was tailored to a ProS audience, refined, and piloted. Ongoing work through ACCESS+ will support engagement and continued refinement through use with future cohorts of ACCESS+ ProSs and the development of complementary resources.

Given the parallel role that mental models play in university and ProS systems, we propose that it is valuable to adapt the *IPF* for use in ProSs. Like the *IPF: Faculty*, the *IPF: Societies* at its core focuses on building an equity mindset through identity awareness and intercultural mindfulness and then puts that mindset into practice through reinforcing skills that support inclusive relationships. Where the frameworks (i.e., *IPF: Faculty and IPF: Societies*) differ is in the contexts and roles of those applying the framework. The *IPF: Faculty* was developed to promote inclusive skill development for faculty across their roles within academic institutions (e.g., teaching, advising, research mentoring, collegiality, and leadership) (Gillian-Daniel et al., 2021b; Dukes et al., in press). For the *IPF: Societies*, application occurs, initially by society DEI change leaders (i.e., “boundary spanners”), in the various functions that the society performs for its members and discipline, as discussed in greater detail below (see Figure 1: *IPF: Societies* with example ProS functions).

The *IPF: Societies* has dual target audience foci: 1) DEI change leaders (individual focus) and 2) the ProSs as a system (organizational focus). Key individuals within the organizational system are ProS DEI change leaders, known as “boundary spanners,” who are people within an organization who work to connect ideas, resources, and stakeholders (Hill, 2020). These individuals engage in five key behaviors: 1) finding—identifying knowledge and resources outside one's organization to advance innovation, research, and development (Ancana and Caldwell, 1992; Tushman and Scanlan, 1981); 2) translating—making sense of what is found for modification and application within one's own organization (Katz and Tushman, 1981); 3) diffusing—sharing what is gained from extra-institutional connections with fellow organizational members (Rogers, 2003); 4) gaining support—laying the political foundation and support within an organization to implement innovation (Brion et al., 2012; Faraj and Yan, 2009); and 5) social “weaving” behaviors by being the bridge wherewith to connect diverse stakeholders from multiple organizations under a common purpose (Burt, 1992; Kania and Kramer, 2011). Boundary spanners are an ideal lever for enacting and promoting DEI change given that they are often in positions to reach other boundary spanners in their ProSs and beyond (Aldrich and Herker, 1977; Katz and Tushman, 1981; Ancona and Caldwell, 1992; Hill, 2020). We propose that uptake of the *IPF: Societies* by boundary spanners to develop and refine DEI awareness, knowledge, and skills can better position these change leaders to make systemic changes within their ProS. This in turn has potential ripple effects extending to the wider STEM system (Leibnitz et al., 2021). Similarly, by STEM ProSs using the *IPF: Societies* to explore the ProS organizational system, both internal-focus (i.e., the STEM ProS business infrastructure) and external-focus (i.e., member and disciplinary serving STEM ProS infrastructure) DEI awareness and organizational capacity are enhanced, better positioning ProSs to enact DEI systems change.

Figure 1 depicts the progression of the *IPF: Societies'* processes, showing how the equity mindset is developed and expands into relationships and actions that guide ProS core functioning, catalyzing STEM DEI systems change. We propose that the *IPF: Societies* can be usefully applied at both individual and organizational levels. Below we describe specific aspects of the *IPF: Societies* as well as its application.

Identity awareness is an awareness of aspects of one's own social and cultural identities and how those identities are situated within larger intersecting systems of power. Intercultural mindfulness is the “ability to understand cultural differences in ways that enable one to interact effectively with others from different racial, ethnic, or social identity groups in both domestic and international contexts” (Gillian-Daniel et al., 2021a). Collectively, “these domains encompass many features of intercultural humility, including: 1) awareness of one's own cultural backgrounds, including intersecting social identities; 2) recognizing one's biases and privileges in relation to self and others; 3) committing to learning about others' cultural backgrounds; and 4) addressing disparities in relational power by, in part, learning to recognize power differentials” (Gillian-Daniel et al., 2021a). The more aware one is of aspects of one's own social and cultural identities, the identities of others, and how those identities are situated within larger, intersecting systems of power, the more equitably mindful one can be of impacts, decisions, and programming driven by those identities.

Equity mindedness underpins building inclusive relationships. At both personal and organizational levels, willingness, capacity, and the communication skills to effectively engage those whose lived experiences may not match one's own is vital for examining mental models and advancing inclusive ProS DEI reform. At the boundary spanner level, inclusive relationships mean reflecting on whose voices are, and are not, centered and carry decision-making power when discussing important ProS policies, processes, and activities. From the STEM ProS perspective, building inclusive relationships could be reflected in collaborations with a range of organizations with intention to build mutual capacity. Inclusive relationships at the society level help shift social narratives and can inform sense making around information collected about the ProS, two examples of how mental models have critical impact on organizational systems (Kania et al., 2018).

Influential actions are how boundary spanners and ProSs drive STEM system change. We propose that informed and diversely networked people serving as DEI boundary spanners will be motivated and held accountable for positive DEI change. Boundary spanners' actions can be focused on core ProS functions. Peters and others (2021) identified 11 functions of STEM ProSs for action focus. For explanatory purposes, we focus on a subset of five ProS functions identified by Peter et al. (2021) as depicted in the outer circles of Figure 1 and highlighted in Table 1. Ultimately, we propose that *IPF: Societies*-informed boundary spanners will engage in the influential actions associated with establishing new mental models and create accountability for nurturing the new diverse, equitable, and inclusive ProS look, feel, and actions.

**TABLE 1:** How the IPF: Societies informs practices within five example professional society functions.

Example ProS function name and definition (Peters et al., 2021)	Example ProS policies/practices	Example ProS questions generated with an *IPF: Societies* lens	Example ideas for implementing more equitable practices
Governance and leadership—How the ProS is run and major decisions are made (internal focus)	Governing board members are selected based on seniority within the discipline	• How is seniority a result of structural inequality within the ProS and U.S. broader society?	• Develop a mission/vision statement or other commitment to equality and diversity that includes a non-discrimination clause regarding leadership and members (e.g., Potvit et al., 2018)
		• How does using seniority as a measure of qualification shape the pool of possible governing board members?	• Identify clear goals and adequate resources to support change
			• Gather inclusive organizational data; analyze the data intersectionally; share results publicly; and use the data to inform action planning
			• Ensure that DEI commitment is reflected consistently in charges to all committees
			• Build a case for more diverse senior leadership as essential to the long-term success of the organization
			• Build understanding, buy-in, and support from grassroots organizational members as well as from leadership
			• Engage male and/or majority member allies and advocates at all levels of the organization in the culture-change effort (e.g., Bilimoria et al., 2008)
Membership—ProS members and the structures that shape membership makeup (external focus)	In order to reduce survey burden and avoid being too intrusive, the ProS collects limited demographic data through its membership application	• What data are collected, and for what purpose(s)	• Maintain accountability by collecting data on society membership and leadership and present these numbers publicly
		• How are the data collected currently used to further an inclusive mission of the society?	• Develop a mission/vision statement or other commitment to equality and diversity that includes a non-discrimination clause regarding leadership and members (e.g., Potvit et al., 2018)
		• Do members feel that the measures accurately capture their social and cultural identities?	• Frame diversity as a positive and enact anti-discriminatory policies (e.g., societal codes of conduct)
		• How is the rationale for collecting demographic data articulated to members as being both valuable and aligned with ProS DEI priorities and efforts?	• Work to address systemic bias to create a culture of belonging and an environment that recognizes and supports the experiences of members with marginalized identities (e.g., Abernethy et al., 2020)
Convenings—Who, where, and how people participate in ProS events (external focus)	Conference committees are composed of volunteers who determine the speakers, program, content, and social activities	• How do social and cultural identities of the committee members affect decisions about speakers, program content, or social activities?	• Switch to fully virtual conferences with multi-location in-person “local” conferences (e.g., Sarabipour et al., 2021)
		• How does the ProS create buy-in from membership around DEI-focused programming?	• Select meeting locations that will be safe for all members
			• Choose environmentally responsible accommodation near public transportation
		• How does the selection of the event's location reflect dominant views about what feels comfortable, safe, or enjoyable (e.g., restaurants, entertainment, amenities)?	• Choose sustainable food catering
			• Provide free and on-site nursing and childcare facilities at regional meetings; include this information in registration materials
			• Generate meeting codes of conduct and ethics (e.g., Sarabipour et al., 2020)
Recognition—The established procedures in which people apply or are nominated for recognition or support (internal and external foci)	Institutional affiliation is required on membership applications, award nominations, and presentation proposals	• How is institutional affiliation tied to structural inequality?	• Broaden what applicant qualifications are considered when awards and recognition are determined. For example, for travel awards, consider both evidence of a candidate's scientific achievement as well as their expressed interest in attending/benefiting from the event
		• Is using institutional affiliation necessary?	
		• Does institutional affiliation serve as a proxy for exclusionary notions of legitimacy, excellence, and thus bias selection?	
		• How are scholars in career transition and without institutional affiliation provided access to ProS resources?	• Vette top nominees by cross-checking code of conduct reports with other societies and contacting Title IX offices at current and previous institutions or employers (e.g., Fernandes et al., 2020)
			• Evaluate the extent to which award program goals and outcomes are being met (e.g., Segarra et al., 2020a)
Professional development—Job boards, mentoring, practitioner continuing education, and similar efforts aimed at cultivating members' successful careers (external focus)	Professional development offerings provide suggestions to members about how to be successful job candidates	• What are the biases or assumptions in career training that reinforce and normalize whiteness and masculinity?	• Provide professional development programming to build core equity, diversity, and inclusion competencies, including and not limited to building awareness around implicit bias (e.g., Coe et al., 2019)
		• What systems can be introduced to improve these society offerings?	
			• Include diversity-related programming during annual meetings (e.g., offer workshops on effective mentoring) (e.g., Abernethy et al., 2020)

## Discussion

The *IPF: Societies* complements the use of other DEI organizational tools and increases both individual and organizational capacity to more efficiently and effectively identify and engage with DEI actions resulting from use of these tools. For example, we offer the Women in Engineering ProActive Network's (WEPAN's) *Four Frames for Promoting Gender Equity Within Organizations* (WEPAN, 2013). Originally adopted from Simmons University's Center for Gender in Organizations (1998), the four frames include: 1) equipping the individual, 2) creating equal opportunity, 3) valuing difference, and 4) revisioning culture. A STEM ProS DEI boundary spanner employing the *IPF: Societies* can evaluate and introduce more inclusive professional development programs (Frame 1); examine and recommend DEI changes to organizational structures, policies, and practices (Frame 2); call attention to ways in which ProS leaders and the organization are not “walking the DEI walk” (Frame 3); and identify and remedy incongruences between ProS existing practices and goals outlined in the ProS strategic plan (Frame 4). Similarly, from an organizational perspective, WEPAN's frames could be used to evaluate the equity of professional development programs and educational pathways (Frame 1); examine and revise organizational structures, policies, and practices to support greater DEI integration across all society functions (Frame 2); ensure that all leaders are, and continue to be, trained and coached on how to enact DEI-focused changes (Frame 3); and create opportunities to re-vision ProS culture and reflect that updated vision in the ProS mission and strategic plans (Frame 4).

As with WEPAN's four frames, the *IPF: Societies* complements the *Equity Environmental Scanning Tool (EEST)* (Peters et al., 2021). The *EEST* is a DEI self-assessment tool for ProSs adapted by ACCESS+ from The Royal Academy of Engineering and Science Council Diversity and Inclusion Progression Framework (2021). We propose that boundary spanners skilled in using the *IPF: Societies* will be more efficiently and effectively able to enact changes in areas identified by the *EEST*. Table 1 illustrates how the *IPF: Societies* can inform ProS DEI practices in relation to a subset (i.e., 5 of the original 11) of Peters et al. (2021) ProS's core functions, each of which have an internal focus (i.e., the STEM ProS business infrastructure) and/or an external focus (i.e., member and disciplinary serving STEM ProS infrastructure). We propose that taking an *IPF: Societies* lens to the policies and practices associated with each of these functions will help uncover and offer an opportunity to change previously implicit ProS mental models. We use questions to illustrate application of the *IPF: Societies*. In each core ProS function (column 1), existing policies or practices are presented that might appear reasonable to some (column 2), but when the *IPF: Societies* lens is applied (column 3), systemic and structural inequities affecting how the ProS engages with staff and members become more visible. We offer example ideas of equitable practices that could emerge from application of the *IPF: Societies* (column 4). This table shows how the ProS may not be making programming decisions with an understanding of structural issues (i.e., equity mindset), therefore missing out on the opportunity to address them and counter obstacles to DEI through inclusive relationships and influential actions.

When and where the *IPF: Societies* is brought into the ProS DEI change cycle will likely be dictated by the culture of the ProS and/or ProS leaders. Examples for how the IPF: Societies can be used and inform engagement is depicted in Table 2 below.

**TABLE 2:** Example IPF: Societies implementation strategies within professional societies.

Level of ProS DEI engagement (Peters, et al., 2021)	Description of DEI engagement level within a society (Peters et al., 2021)	Example IPF: Society-based implementation strategies
No activity	No case for DEI has been developed yet	Society boundary spanners use the IPF: Societies to help identify a network of others interested in DEI change and make the case for DEI engagement to ProS leaders and members.
Idling	The DEI case is developing; however, DEI has not been prioritized; no substantial planning or activity	Society boundary spanners engage in IPF: Societies-based programming to build their equity mindset and interpersonal communication skills.
Emerging	There is a DEI case for action; initial DEI conversations, planning, and action are underway, and activity is minimal/ad hoc	Society boundary spanners use the framework together with a DEI tool to work with leadership and staff to identify areas of opportunity for growth in the society. Their equity mindset supports them asking equity-based questions about society functions. Society policies and procedures are considered through this lens.
Progressing	The DEI case is well established; DEI actions have been carried out from planning phases, and activity may not be routine yet	Society boundary spanners work with staff and key members to design and implement DEI-based programming. Collaborations with other organizations and initiatives allow the society to leverage existing programming and resources as they infuse DEI throughout the society. There is a “tipping point” of engagement by leadership, staff, and now membership in these programs that support the “institutionalization” of said efforts.
Achieving	The DEI case is being realized; planning and action have been underway for several iterations, and impacts are clear	Society leadership and staff routinely collect and review data, for example, on membership, about who engages in society leadership, on who speaks at society functions, and who receives recognition from the society for their scholarship. Policies and procedures are regularly reviewed and revised to be more equitable and inclusive. The society uses a DEI tool to benchmark their progress relative to peer societies and collaborates with these societies to share best practices.

## Conclusion

In sum, Identity awareness and Intercultural mindfulness create an equity mindset that supports inclusive relationships and influential actions. The four “I's” core to the *IPF: Societies* provide a framework for reflecting and acting on ProS culture at individual (e.g., STEM ProS DEI boundary spanner) and organizational levels. The *IPF: Societies* offers a way to guide change of mental models. ProS DEI boundary spanners employing the *IPF: Societies* can leverage their positionality and ability to straddle groups to affect cultural change across STEM ProSs, in combination with the efforts of other boundary spanners and in the disciplines in which they engage.

Of critical importance when working with mental models in ProSs is the expectation that there may be resistance to DEI initiatives, especially among members with majoritized identities who may be invested, even subconsciously, in maintaining existing power structures (Lipsitz, 2006). Because people occupy a constellation of identities of various positionings, awareness of common discourses rejecting DEI could help in ProSs navigating them (Bonilla-Silva, 2006). The *IPF: Societies* offers a framework to begin difficult discussions and offers a structured approach for working toward change. Of course, to be effective, the *IPF: Societies* requires sustained mobilization of its pieces, *vis-à-vis* making DEI concerns part of the fabric of ProSs.

Potential outcomes of wide-scale implementation of the *IPF: Societies* could be ProS actions in service of a more diverse, inclusive, and equitable STEM culture writ large. Resultant increased individual capacity to engage in the articulation and reframing of legacy mental models in turn guides organizational transformation and culture reform through broader systems change. As organizations engage in systemic change, greater ProS and STEM culture DEI changes can be made. Eventually, DEI change becomes less about individual efforts for specific DEI actions and more about broad, structurally patterned ProS organizational transformation and, ultimately, STEM culture reform.

## Data Availability

The original contributions presented in the study are included in the article/Supplementary Material; further inquiries can be directed to the corresponding author.

## References

[B1] Abernethy E. F. Arismendi I. Boegehold A. G. Colón-Gaud C. Cover M. R. Larson E. I. (2020). Diverse, Equitable, and Inclusive Scientific Societies: Progress and Opportunities in the Society for Freshwater Science. Freshw. Sci. 39 (3), 363–376. 10.1086/709129

[B2] Aldrich H. Herker D. (1977). Boundary Spanning Roles and Organization Structure. Amr 2 (2), 217–230. 10.5465/amr.1977.4409044

[B3] Amplifying the Alliance to Catalyze Change for Equity in STEM Success (ACCESS+) (n.d.). Retrieved from https://accessplusstem.com/ (Accessed September 22, 2021). 10.1128/jmbe.00340-21 PMC8941930 35340448

[B4] Ancona D. G. Caldwell D. F. (1992). Bridging the Boundary: External Activity and Performance in Organizational Teams. Administrative Sci. Q. 37 (4), 634–665. 10.2307/2393475

[B5] Baillie C. Kabo J. Reader J. (2012). Heterotopia: Alternative Pathways to Social justice. Zero Books.

[B6] Bilimoria D. Joy S. Liang X. (2008). “Breaking Barriers and Creating Inclusiveness: Lessons of Organizational Transformation to advance Women Faculty in Academic Science and Engineering,”. Hum. Resour. Manage. 47, 423–441. 10.1002/hrm.20225

[B7] Bonilla-Silva E. (2006). Racism without Racists: Color-Blind Racism and the Persistence of Racial Inequality in the United States. Rowman & Littlefield Publishers.

[B8] Brion S. Chauvet V. Chollet B. Mothe C. (2012). Project Leaders as Boundary Spanners: Relational Antecedents and Performance Outcomes. Int. J. Project Manag. 30, 708–722. 10.1016/j.ijproman.2012.01.001

[B9] Burt R. S. (1992). Structural Holes: The Social Structure of Competition. Harvard University Press.

[B10] Campbell-Montalvo R. A. Caporale N. McDowell G. S. Idlebird C. Wiens K. M. Jackson K. M. (2020). Insights from the Inclusive Environments and Metrics in Biology Education and Research Network: Our Experience Organizing Inclusive Biology Education Research Events. J. Microbiol. Biol. Educ. 21 (1), 1–9. 10.1128/jmbe.v21i1.2083 PMC7195160 32431765

[B11] Campbell-Montalvo R. Kersaint G. Smith C. Puccia E. Sidorova O. Cooke H. (in press). The Influence of Professional Engineering Organizations on Women and Underrepresented Minority Students' Fit. Front. Edu.

[B12] Campbell-Montalvo R. Kersaint G. Smith C. Puccia E. Skvoretz J. Wao H. (2021). How Stereotypes and Relationships Influence Women and Underrepresented Minority Students' Fit in Engineering. J. Res. Sci. Teach., 1–37. 10.1002/tea.21740

[B13] Cech E. A. Rothwell W. R. (2018). LGBTQ Inequality in Engineering Education. J. Eng. Educ. 107 (4), 583–610. 10.1002/jee.20239 PMC10544662 37786450

[B14] Chaudhary B. Berhe A. A. (2020). Ten Simple Rules for Building an Anti-racist Lab. Charlottesville, VA: Center for Open Science. 10.32942/osf.io/4a9p8

[B15] Coe I. R. Wiley R. Bekker L. G. (2019). Organisational Best Practices towards Gender equality in Science and Medicine. Lancet 393 (10171), 587–593. Available at:https://www.sciencedirect.com/science/article/pii/S014067361833188X . 10.1016/S0140-6736(18)33188-X 30739694

[B16] Collins P. H. (2015). Intersectionality's Definitional Dilemmas. Annu. Rev. Sociol. 41, 1–20. 10.1146/annurev-soc-073014-112142

[B17] Crenshaw K. (1989). Demarginalizing the Intersection of Race and Sex: A Black Feminist Critique of Antidiscrimination Doctrine, Feminist Theory and Antiracist Politics, 139. Chicago: University of Chicago Legal Forum.

[B18] Crenshaw K. (1991). Race, Gender, and Sexual Harassment. South. Calif. L. Rev. 65, 1467.

[B19] Dukes A. A. Gillian-Daniel D. L. Greenler R. Mc. C. Parent R. A. Bridgen S. Esters L. T. (In press). “The Aspire Alliance Inclusive Professional Framework for Faculty—Implementing Inclusive and Holistic Professional Development that Transcends Multiple Faculty Roles,” in The Handbook of STEM Faculty Development. Editors Linder S. Lee C. High K. (Charlotte, NC: American Society for Engineering Education).

[B20] Eisenhart M. A. Finkel E. (1998). Women's Science: Learning and Succeeding from the Margins. Chicago: University of Chicago Press.

[B21] Etson C. Block K. Burton M. D. Edwards A. Flores S. Fry C. (2021). Beyond Ticking Boxes: Holistic Assessment of Travel Award Programs Is Essential for Inclusivity. Charlottesville, VA: OSF Preprints 10.31219/osf.io/fsrpb

[B22] Faraj S. Yan A. (2009). Boundary Work in Knowledge Teams. J. Appl. Psychol. 94 (3), 604–617. 10.1037/a0014367 19450002

[B23] Faulkner W. (2007). ‘Nuts and Bolts and People’. Soc. Stud. Sci. 37 (3), 331–356. 10.1177/0306312706072175

[B24] Fernandes A. M. Abeyta A. Mahon R. C. Martindale R. Bergmann K. D. Jackson C. A. (2020). “Enriching Lives within Sedimentary Geology”: Actionable Recommendations for Making SEPM a Diverse. Equitable and Inclusive Society for All Sedimentary Geologists. Pre-printAvailable at: https://eartharxiv.org/repository/object/90/download/170/ .

[B25] Foor C. E. Walden S. E. Trytten D. A. (2007). "I Wish that I Belonged More in This Whole Engineering Group:" Achieving Individual Diversity. J. Eng. Edu. 96 (2), 103–115. 10.1002/j.2168-9830.2007.tb00921.x

[B26] Forrester N. (2020). Diversity in Science: Next Steps for Research Group Leaders. Nature 585, S65–S67. 10.1038/d41586-020-02681-y

[B27] Foucault M. (2007). Discipline and Punish: The Birth of the Prison. Duke University Press.

[B28] Gillian-Daniel D. L. Troxel W. G. Bridgen S. (2021b). Promoting an Equity Mindset through the Inclusive Professional Framework for Faculty. The Department Chair 32 (2), 4–5. 10.1002/dch.30408

[B29] Gillian-Daniel D. L. Greenler R. M. McCBridgen S. T. Bridgen S. T. Dukes A. A. Hill L. B. (2021a). Inclusion in the Classroom, Lab, and beyond: Transferable Skills via an Inclusive Professional Framework for Faculty. Change Mag. Higher Learn. 53 (5), 48–55. 10.1080/00091383.2021.1963158

[B30] Hacker S. L. (1989). Pleasure, Power and Technology: Some Tales of Gender, Engineering, and the Cooperative Workplace. Boston, MA: Unwin Hyman.

[B31] Hacker S. L. (1981). The Culture of Engineering: Woman, Workplace and Machine. Women's Stud. Int. Q. 4 (3), 341–353. 10.1016/s0148-0685(81)96559-3

[B32] Hill L. B. (2020). Understanding the Impact of a Multi-Institutional STEM Reform Network through Key Boundary-Spanning Individuals. J. Higher Edu. 91 (3), 455–482. 10.1080/00221546.2019.1650581

[B33] Hughes B. E. (2018). Erratum for the Research Article: "Coming Out in STEM: Factors Affecting Retention of Sexual Minority STEM Students" by B. E. Hughes. Sci. Adv. 4 (3), eaau2554. 10.1126/sciadv.aao637310.1126/sciadv.aau2554 30079378 PMC6068014

[B34] INCLUDES Aspire Alliance National Change(n.d). Inclusive Professional Framework for Societies. Available at: https://www.aspirealliance.org/national-change/inclusive-professional-framework/ipf-societies (Accessed on September 22, 2021)

[B35] Griffin K. A. Museus S. D. (Editors) (2011). Using Mixed-Methods to Study Intersectionality in Higher Education: New Directions in Institutional Research (Jossey-Bass).

[B36] Kania J. Kramer M. (2011). Collective Impact. Stanford Soc. Innovation Rev. 9 (1), 36–41. Available at: https://ssir.org/articles/entry/collective_impact .

[B37] Kania J. Kramer M. Senge P. (2018). The Water of Systems Change. FSG. Available at: http://efc.issuelab.org/resources/30855/30855.pdf .

[B38] Katz R. Tushman M. (1981). An Investigation into the Managerial Roles and Career Paths of Gatekeepers and Project Supervisors in a Major R & D Facility. R. D Manag. 11 (3), 103–110. 10.1111/j.1467-9310.1981.tb00458.x

[B39] Borman K. Halperin R. Tyson W. (Editors) (2010). Becoming an Engineer in Public Universities: Pathways for Women and Minorities (New York, NY: Springer).

[B40] Leibnitz G. M. Gillian-Daniel D. L. Hill L. B. (2021). Networking Networks: Leveraging STEM Professional Society “Boundary Spanners” to Advance Diversity, Equity, and Inclusion. NSF INCLUDES Rapid Community ReportsAvailable at: https://adobe.ly/3fPxjUs .

[B41] Lipsitz G. (2006). The Possessive Investment in Whiteness: How white People Profit from Identity Politics. Philadelphia, PA: Temple University Press.

[B42] Lohan M. Faulkner W. (2004). Masculinities and Technologies. Men and masculinities 6 (4), 319–329. 10.1177/1097184x03260956

[B43] McGee E. O. (2020). Black, Brown, Bruised: How Racialized STEM Education Stifles Innovation. Cambridge, MA: Harvard Education Press.

[B44] Metcalf H. (2016). Broadening the Study of Participation in the Life Sciences: How Critical Theoretical and Mixed-Methodological Approaches Can Enhance Efforts to Broaden Participation. Lse 15 (3), rm3. 10.1187/cbe.16-01-0064 PMC5008906 27521238

[B45] Metcalf H. Russell D. Hill C. (2018). Broadening the Science of Broadening Participation in STEM through Critical Mixed Methodologies and Intersectionality Frameworks. Am. Behav. Scientist 62 (5), 580–599. 10.1177/0002764218768872

[B46] Metcalf H. (2017). Science Must Clean up its Act. Scientific American. Available at: https://blogs.scientificamerican.com/voices/science-must-clean-up-its-act/ .

[B47] National Academy of Sciences (2005). National Academy of Engineering, and Institute of MedicineFacilitating Interdisciplinary Research. Washington, DC: The National Academies Press. 10.17226/11153

[B48] Page S. (2007). The Difference: How the Power of Diversity Creates Better Groups, Firms, Schools, and Societies. Princeton, NJ: Princeton University Press.

[B49] Page S. (2017). The Diversity Bonus. Mellon Foundation: Princeton University Press and Andrew W.

[B50] Pawley A. Tonso K. L. (2011). Monsters of Unnaturalness: Making Women Engineers' Identities via Newspapers and Magazines (1930-1970). J. Soc. Women Eng. 20, 50–75.

[B51] Peters J. Campbell-Montalvo R. Leibnitz G. Metcalf H. Lucy-Putwen A. Gillian-Daniel D. (2021). Refining an Assessment Tool to Optimize Gender Equity in Professional STEM Societies. WCER Working Paper No. 2021-7 Available at: https://wcer.wisc.edu/docs/working-papers/WCER_Working_Paper_No_2021_7.pdf . 10.3389/fsoc.2022.755372 PMC9237367 35774108

[B52] Potvin D. A. Burdfield-Steel E. Potvin J. M. Heap S. M. (2018). Diversity Begets Diversity: A Global Perspective on Gender equality in Scientific Society Leadership. PloS one 13 (5), e0197280. Available at: https://journals.plos.org/plosone/article?id=10.1371/journal.pone.0197280 . 10.1371/journal.pone.0197280 29847591 PMC5976142

[B53] Riley D. Slaton A. E. Pawley A. L. Johri A. Olds B. M. (2014). Social justice and Inclusion: Women and Minorities in Engineering. Cambridge handbook Eng. Educ. Res., 335–356.

[B54] Rogers E. M. (2003). Diffusion of Innovations. 5th ed. New York, NY: Free Press.

[B55] Sarabipour S. Khan A. Seah Y. F. S. Mwakilili A. D. Mumoki F. N. Sáez P. J. (2021). Changing Scientific Meetings for the Better. Nat. Hum. Behav. 5 (3), 296–300. Available at: https://www.nature.com/articles/s41562-021-01067-y . 10.1038/s41562-021-01067-y 33723404

[B56] Sarabipour S. Schwessinger B. Mumoki F. N. Mwakilili A. D. Khan A. Debat H. J. (2020). Evaluating Features of Scientific Conferences: A Call for Improvements. BioRxiv. Available at: https://www.biorxiv.org/content/biorxiv/early/2020/04/21/2020.04.02.022079.full.pdf .

[B57] Segarra V. A. Primus C. Unguez G. A. Edwards A. Etson C. Flores S. C. (2020b). Scientific Societies Fostering Inclusivity through Speaker Diversity in Annual Meeting Programming: a Call to Action. Mol. Biol. Cel 31 (23), 2495–2501. 10.1091/mbc.E20-06-0381 PMC7851875 33119460

[B58] Segarra V. A. Vega L. R. Primus C. Etson C. Guillory A. N. Edwards A. (2020a). Scientific Societies Fostering Inclusive Scientific Environments through Travel Awards: Current Practices and Recommendations. CBE Life Sci. Educ. 19 (2), es3. 10.1187/cbe.19-11-0262 32453676 PMC8697665

[B59] Seymour E. Hewitt N. M. (1997). Talking about Leaving. Boulder, CO: Westview Press.

[B60] Skvoretz J. Kersaint G. Campbell-Montalvo R. Ware J. D. Smith C. A. S. Puccia E. (2020). Pursuing an Engineering Major: Social Capital of Women and Underrepresented Minorities. Stud. Higher Edu. 45 (3), 592–607. 10.1080/03075079.2019.1609923

[B61] Smith C. A. S. Wao H. Kersaint G. Campbell-Montalvo R. Gray-Ray P. Puccia E. (2021). Social Capital from Professional Engineering Organizations and the Persistence of Women and Underrepresented Minority Undergraduates. Front. Sociol. 6, 1–13. 10.3389/fsoc.2021.671856 PMC8203332 34136561

[B62] The Royal Academy of Engineering and the Science Council (2021). Diversity Progression Framework 2.0 for Professional Bodies: A Framework for Planning and Assessing Progress. Retrieved from: https://sciencecouncil.org/professional-bodies/diversity-equality-and-inclusion/diversity-framework/ (Accessed on September 22, 2021).

[B63] Tonso K. L. (1999). Engineering Gender− Gendering Engineering: A Cultural Model for Belonging. J. women minorities Sci. Eng. 5 (4), 365–405. 10.1615/jwomenminorscieneng.v5.i4.60

[B64] Tonso K. L. (2014). Making Science Worthwhile: Still Seeking Critical, Not Cosmetic, Changes. Cult. Stud. Sci. Educ. 9 (2), 365–368. 10.1007/s11422-012-9448-5

[B65] Tonso K. L. (2007). On the Outskirts of Engineering: Learning Identity, Gender, and Power via Engineering Practice. Boston, MA: Brill.

[B66] Tonso K. L. (1996). The Impact of Cultural Norms on Women*. J. Eng. Edu. 85 (3), 217–225. 10.1002/j.2168-9830.1996.tb00236.x

[B67] Trouillot M. R. (1995). Silencing the Past: Power and the Production of History. Beacon Press.

[B68] Tushman M. L. Scanlan T. J. (1981). Boundary Spanning Individuals: Their Role in Information Transfer and Their Antecedents. Amj 24 (2), 289–305. 10.5465/255842

[B70] Warner L. R. Settles I. H. Shields S. A. (2016). Invited Reflection. Psychol. Women Q. 40 (2), 171–176. 10.1177/0361684316641384

[B71] Women in Engineering ProActive Network (WEPAN). (2013). Framework for Promoting Gender Equity in Organizations. Available at: https://www.wepan.org/general/custom.asp?page=FourFrames (Accessed September 22, 2021).

